# Research Note: Corn energy and nutrient utilization by broilers as affected by geographic areas and carbohydrases

**DOI:** 10.1016/j.psj.2022.102366

**Published:** 2022-11-30

**Authors:** Catarina Stefanello, Sergio Luiz Vieira, Heitor Vieira Rios, Patricia Soster, Cristina Tonial Simoes, Guilherme Godoy, Vitor Fascina

**Affiliations:** ⁎ Department of Animal Science, Federal University of Santa Maria, Santa Maria, RS, Brazil; † Department of Animal Science, Federal University of Rio Grande do Sul, Porto Alegre, RS, Brazil; ‡ DSM Nutritional Products S.A., São Paulo, SP, Brazil

**Keywords:** amylase, digestibility, endosperm, starch, xylanase

## Abstract

Two experiments (**Exp.**) were conducted to evaluate the effects of exogenous carbohydrases on nutrient and energy utilization of corn with different compositions by broilers. In Exp. 1, a total of 448 Cobb 500 male chicks were distributed in a 2 × 4 factorial arrangement (corn from regions geographically located in the North or South of Brazil × 4 carbohydrases supplementation), with 8 replicate cages of 7 birds each. In Exp. 2, 672 Cobb 500 male chicks were fed 12 experimental feeds, in a 3 × 4 factorial arrangement [3 corn endosperm compositions (waxy, semi-dent, or semi-flint) × 4 carbohydrases], with 8 replicate cages of 7 birds. Birds were fed semi-purified test diets with 95.9% corn from d 14 to 24 in both studies. In Exp. 1, α-amylase, β-xylanase, or carbohydrase complex (cellulase, glucanase, and xylanase) were added to the diet. In Exp. 2, α-amylase, β-xylanase, or α-amylase + β-xylanase were supplemented. Digestibility of DM, N, ether extract (**EE**), Ca, and P as well as AME, AME_n_, and IDE were determined. In Exp. 2, jejunal starch digestibility was determined on d 24. Data were subjected to analysis of variance and means were compared by Tukey test (*P* ≤ 0.05). Corn from the North origin had higher AME, AME_n_, and digestibility of DM and N compared to the South (*P* ≤ 0.05). Amylase supplementation led to increases of 3% in AME and 2% in N digestibility when compared to the non-supplemented feeds (*P* ≤ 0.01). In Exp. 2, the highest ME values and EE digestibility were observed in the semi-flint corn compared to the waxy, whereas the semi-dent presented the highest digestibility of N and starch. Corn diets supplemented with amylase + xylanase had improvements of 2.5% AME_n_ and 3% starch digestibility. In conclusion, energy and nutrient utilization of corn by broilers depend on the region where it was grown. Corn genetics, expressed by the endosperm composition, and carbohydrase supplementation influenced energy and nutrient utilization by broilers.

## INTRODUCTION

Corn (*Zea mays* L.) is the major energy-yielding source in poultry feeds worldwide, and its contribution averages 65% of bird metabolizable energy allowances throughout growth. Investments in ingredient analyses in feed mills have become more useful since there is an understanding that diet composition can be variable according to ingredient variability. For instance, corn nutrient values vary depending on the contents of starch, protein, fat, and non-starch polysaccharides (**NSP**) as well as resistant starch (Cowieson, 2005).

Arabinoxylans are predominant in the corn endosperm cell walls, and small amounts of mixed-linked β-glucan, xylans, and cellulose are found in the cell walls of its hull fraction. Resistant starch, which is not well digested by broilers, may account from 2 to 6% of the total starch depending on the corn source (Weurding et al., 2001). Nutrients and energy from corn may not be completely digested in the small intestine of chickens since physical barriers exist and prevent the access to bird endogenously secreted enzymes. Therefore, foreseen existing opportunities for the addition of carbohydrases in corn-based diets targeting those low digestibility substrate fractions. This is expected to lead to an increase in energy and overall nutrient utilization.

In the commercial practice, responses to enzyme supplementation in broiler feeds vary depending on factors such as supplemental enzyme activity, specificity and interactions between enzymes, ingredient quality, and type, as well as substrate concentration, level of nutrients in the diet, and age of birds. Corn composition differences have been related to corn genetics, soil characteristics, and field conditions that can be affected by geographic areas (Cowieson and Ravindran, 2008).

The objective of the present study was to evaluate the effects of corn originated from different genetics and growth environments on energy and nutrient digestibility by broiler chickens. Additionally, these effects were evaluated in corn-based diets supplemented with exogenous amylase, xylanase, or glucanase.

## MATERIALS AND METHODS

All procedures used in this study were approved by the Ethics and Research Committee of the Federal University do Rio Grande do Sul, Porto Alegre, RS, Brazil.

### Broiler Husbandry

A total of 1,120 slow-feathering Cobb 500 one-day-old male broilers (45 ± 1.0 g), vaccinated for Marek's disease at a commercial hatchery (BRF, Lajeado, RS, Brazil) were randomly placed into wire cages in a temperature-controlled room. Each cage was equipped with one trough feeder (0.4 m length) and one drinker, which allowed ad libitum access to water and mash feeds. Average temperature was 32°C at placement, being reduced by 1°C every 2 d until 23°C to provide comfort throughout the study. Lighting was continuous until d 24.

In experiment (**Exp.**) 1, a total of 448 birds were reared in 64 cages (0.9 × 0.4 m^2^) and fed 8 experimental diets with 8 replicate cages of 7 birds each in a completely randomized design. In Exp. 2, 672 birds were allocated into 96 cages and fed 12 experimental diets with 8 replicate cages of 7 birds each. In both Exp., standard corn-soy starter diets were fed from d 0 to 14 (3,050 kcal/kg AME, 21.7% CP, 1.05% Ca, and 0.53% non-phytate P). On d 14, chicks were weighed into groups of 7 birds per cage prior to placement. From d 14 to 24, broilers were fed semi-purified mash feeds with 95.9% of corn as the sole source of energy and protein. In Exp. 1, the semi-purified diet used semi-dent corn from 2 regions in Brazil (North, from Mato Grosso state or South, from Rio Grande do Sul state), whereas in Exp. 2 the semi-purified diet had corns from 3 different endosperm types (waxy, semi-dent, or semi-flint). The average of corn particle size in both Exp. was 870 ± 5.0 μm.

### Diets and Experimental Design

Chemical composition and analyzed gross energy (**GE**) of corn were determined in triplicates. The semi-purified diets used in Exp. 1 and 2 had 95.91% corn, 1.31% dicalcium phosphate, 1.12% limestone, 0.51% salt, 0.15% vitamin and mineral premix. Celite at 1% was used as indigestible marker and 1,000 fungal phytase units (**FYT**)/kg of diet [Ronozyme HiPhos (GT)] was included in all diets.

In Exp. 1, a 2 × 4 factorial arrangement of 2 semi-purified basal diets composed by semi-dent corn grown in two regions geographically located in the North or South of Brazil (North or South) and 4 carbohydrases supplementation were used. Corn from each distinct region were cultivated in the same season and, therefore, harvested along a close period of time. The semi-purified basal diets were not supplemented or supplemented with α-amylase, β-xylanase, or a carbohydrase complex that had β-glucanase as the main enzyme activity.

In Exp. 2, a 3 × 4 factorial arrangement was used, with 3 corn endosperms (waxy, semi-dent, or semi-flint) and 4 carbohydrases. The semi-purified diets with waxy, semi-dent, or semi-flint corn types were not supplemented or supplemented with α-amylase, β-xylanase, or α-amylase + β-xylanase mono-component enzyme products.

The α-amylase was a granulated enzyme preparation produced by submerged fermentation of *Bacillus amyloliquefaciens* and contained 600 kilo-Novo α-amylase units (**KNU**)/g (80 KNU/kg feed; Ronozyme HiStarch, Novozymes A/S, Bagsvaerd, Denmark). The activity of 1 KNU is defined as the amount of enzyme that hydrolyses 1 mg of soluble starch/min at 60°C and pH 6.0. The β-xylanase was a granulated heat-stable endo-xylanase from *Thermomyces lanuginosus* produced by submerged fermentation of a genetically modified *Aspergillus oryzae* containing 1,000 fungal xylanase units (**FXU**)/g (100 FXU/kg feed; Ronozyme WX CT, Novozymes A/S, Bagsvaerd, Denmark). One FXU is the amount of endo-1,4-β-xylanase that liberates 7.8 micromoles of reducing sugars (xylose equivalents)/min from azo-wheat arabinoxylans at 50°C and pH 6.0.

The carbohydrase complex used in Exp. 1 was a granulated carbohydrase produced by *Trichoderma longibrachiatum*, containing 8,000 units/g of cellulase, 18,000 units/g of endo-1,3(4)-β-glucanase (**FBG**) and 26,000 units/g of endo-1,4-β-xylanase (60 cellulase units/kg, 135 FBG/kg and 195 endo-1,4-β-xylanase units/kg feed; Roxazyme G2, Novozymes A/S, Bagsvaerd, Denmark). Samples of feeds were analyzed to determine the enzymes recovery.

### Experimental Procedures

In both experiments, excreta samples were collected twice daily on plastic sheets from d 21 to 23, pooled by cage, stored at −20°C, and dried in a forced air oven at 55°C. On d 24, all birds were euthanized and ileal contents were collected. Ileal contents were flushed with distilled water into plastic containers, pooled by cage, immediately frozen in liquid N, and stored at −20°C until lyophilization. In Exp. 2, contents of jejunum were also collected to analyze DM, marker, and starch composition. Triplicate proximate analyses were performed on ground feeds, excreta, and ileal digesta samples. Samples were analyzed for GE, DM, and marker to determine the ileal digestible energy (**IDE**), AME, and AME_n_. Apparent ileal digestibility of DM, N, and ether extract (**EE**) as well as total tract metabolizability of DM, EE, and N were calculated. Analyses, methods, and calculations were done as previously described by Stefanello et al. (2016, 2019).

In Exp. 2, Ca and total P content of the diets and excreta were analyzed (methods 927.02 and 965.17, respectively; AOAC International, 2000). Available, resistant, and total starch levels in the diets and jejunal contents were analyzed using the adapted method 996.11 (AOAC International, 2000) according to Schramm et al. (2021).

### Statistical Analysis

The normal and homogeneous data were submitted to a 2-way ANOVA using the GLM procedure of SAS Institute (version 9.4). Significance was accepted at 5% and mean differences were compared by Tukey's HSD test.

## RESULTS AND DISCUSSION

Analyses of amylase, xylanase, and glucanase in the experimental diets indicated that the supplemental enzymes had activities well-proportioned with the expected values (overall mean of analyzed enzymes in-feed activities were 104 FXU/kg, 82 KNU/kg, and 1,080 FBG/kg).

In Exp. 1, the analyzed composition of corn from the North origin was 3,891 kcal/kg GE, 7.60% CP, 79.2% amylopectin, 64.7% total starch, 3.07% EE, and 0.88% crude fiber (**CF**), while corn from the South origin had 3,855 kcal/kg GE, 7.44% CP, 74.3% amylopectin, 59.3% total starch, 3.63% EE, and 1.07% CF. In Exp. 2, corn classified as semi-flint had 4,043 kcal/kg GE, 7.62% CP, 82.7% amylopectin, 64.2% total starch, 4.10% EE, and 1.16% CF; the semi-dent had 4,082 kcal/kg GE, 7.58% CP, 82.4% amylopectin, 68.0% total starch, 4.31% EE, and 1.30% CF; and the analyzed composition of waxy corn was 4,122 kcal/kg GE, 8.04% CP, 94.2% amylopectin, 68.9% total starch, 4.34% EE, and 1.26% CF.

In Exp. 1, the effects of carbohydrases supplementation on total tract metabolizability and ileal digestibility by broilers fed diets with corn from the 2 distinct regions are presented in Table 1. Corn from the North origin resulted in higher AME (*P* = 0.001) and AME_n_ (*P* = 0.002) values as well as higher digestibility of DM (*P* = 0.004), N (*P* = 0.001), and EE (*P* = 0.003). Corn hybrids are unknown in this study; however, both corn origins had the same endosperm type. The North region produces corn and soybean in favorable climate conditions for agricultural crops and bigger production areas; however, 60% of broiler production is done in the South, demanding corn and soybean meal for the diets.

**Table 1:** Energy and nutrient utilization of corn from different origins not supplemented or supplemented with exogenous carbohydrases, Exp. 1^1^.

Item	Total tract metabolizability					Ileal digestibility		
	AME, kcal/kg	AME_n_, kcal/kg	DM, %	N, %	EE^2^, %	IDE, kcal/kg	DM, %	N, %
Corn origin^3^								
North	3,599	3,465	83.2	66.7	79.7	3,431	77.5	83.0
South	3,524	3,401	82.4	60.6	75.6	3,385	75.7	82.9
Exogenous carbohydrases								
Non-supplemented	3,508^c^	3,384^b^	80.8^b^	60.6^b^	74.9^b^	3,331^b^	74.9^b^	81.2^b^
Amylase^4^	3,615^a^	3,488^a^	83.9^a^	64.2^ab^	79.8^a^	3,456^a^	76.7^ab^	83.2^ab^
Xylanase^5^	3,558^b^	3,424^ab^	83.1^a^	64.1^ab^	77.6^ab^	3,410^ab^	77.1^ab^	83.3^ab^
Carbohydrase complex^6^	3,566^b^	3,437^ab^	83.6^a^	65.6^a^	78.3^ab^	3,436^ab^	77.6^a^	84.1^a^
SEM	9.08	10.91	0.21	0.66	0.60	15.91	0.38	0.30
*P*-value main effects								
Corn origin	0.001	0.002	0.004	0.001	0.003	0.132	0.013	0.903
Carbohydrase	0.001	0.003	0.001	0.005	0.015	0.025	0.043	0.009
Corn origin × carbohydrase	0.089	0.509	0.542	0.091	0.930	0.476	0.866	0.503

a-c Means in the same column with different superscript letter are significantly different by Tukey test (*P* ≤ 0.05).

1 Means (on DM basis) were obtained from 8 replicate cages of 7 birds each.

2 EE = ether extract.

3 Corn from regions geographically located in the North or South of Brazil.

4 Amylase = 80 kilo-Novo α-amylase units (KNU)/kg of feed.

5 Xylanase = 100 β-xylanase units (FXU)/kg of feed.

6 Carbohydrase complex = 60 cellulase units/kg, 135 endo-1,3(4)-β-glucanase units (FBG)/kg, and 195 endo-1,4-β-xylanase units/kg of feed.

Average improvements of 3% AME, 4.9% EE digestibility, and 2% N digestibility were found when corn-based diets were supplemented with amylase (*P* ≤ 0.01) in Exp. 1. The xylanase supplementation resulted in 1.4% AME improvement as well as 2.7% EE digestibility and 2% N digestibility (*P* ≤ 0.01). The AME, DM, and N digestibility were also enhanced when the carbohydrase complex was supplemented to the corn diet when compared to the non-supplemented feed for broilers (*P* ≤ 0.01).

In Exp. 2, the semi-flint corn presented the highest AME, AME_n_, and IDE values as well as the highest EE digestibility of corn for 24-day-old broilers (*P* ≤ 0.05; Table 2). The waxy corn presented higher analyzed GE and starch composition compared to semi-dent and semi-flint corn textures; however, this corn type presented the lowest ME values. The highest digestibility of resistant and total starch in jejunum was observed in the semi-dent corn (*P* = 0.001). The correlation between corn endosperm texture and nutrient digestibility on animal performance has already been evaluated in previous studies (Yegani and Korver, 2013; Tang et al., 2014); however, there is a lack of peer-reviewed data relating corn types, corn nutrient utilization, and carbohydrases for poultry.

**Table 2:** Energy and nutrient utilization of corn with different endosperm textures, not supplemented or supplemented with exogenous amylase, xylanase, or the combination of amylase and xylanase, Exp. 2^1^.

Item	Total tract metabolizability								Ileal digestibility				Jejunal digestibility of starch, %	
	AME, kcal/kg	AME_n_, kcal/kg	DM, %	N, %	EE^2^, %	Ca, %	P, %	IDE, kcal/kg	DM, %	N, %	EE, %	Digestible	Resistant	Total
Corn types														
Waxy	3,445^b^	3,267^b^	76.9^b^	57.3^ab^	63.2^c^	32.1^b^	24.2	3,153^b^	76.9	78.1^ab^	81.6^c^	70.9^a^	72.2^c^	70.1^a^
Semi-dent	3,448^b^	3,281^ab^	77.9^a^	58.2^a^	68.7^b^	34.5^ab^	24.8	3,139^b^	77.1	78.7^a^	87.0^b^	66.7^b^	79.3^a^	68.7^a^
Semi-flint	3,475^a^	3,297^a^	78.3^a^	56.5^b^	75.1^a^	36.9^a^	22.8	3,199^a^	77.0	77.7^b^	88.3^a^	63.9^c^	77.0^b^	65.9^b^
Exogenous carbohydrases														
Non-supplemented	3,418^b^	3,233^b^	77.3^b^	57.1	64.1	33.9	23.2	3,106^b^	76.5^b^	77.1^b^	84.3^b^	64.5^b^	74.1^c^	65.6^b^
Amylase^3^	3,465^a^	3,301^a^	77.6^ab^	57.7	65.9	34.5	24.1	3,184ª	76.8^b^	78.2^ab^	85.3^ab^	67.0^a^	76.4^b^	67.0^a^
Xylanase^4^	3,463^a^	3,297^a^	77.7^ab^	57.5	65.1	34.4	24.0	3,173^a^	76.9^ab^	78.6^a^	86.4^a^	66.2^a^	76.8^b^	68.0^a^
Amylase + xylanase	3,479^a^	3,315^a^	78.2^a^	57.3	65.9	35.0	24.3	3,191^a^	77.7^a^	78.9^a^	86.4^a^	67.3^a^	77.2^a^	68.6^a^
SEM	5.89	6.86	0.14	0.51	1.11	0.48	0.43	8.88	0.16	0.20	0.41	0.46	0.51	0.41
*P*-value main effects														
Corn type	0.049	0.048	0.002	0.001	0.001	0.001	0.147	0.009	0.873	0.112	0.001	0.001	0.001	0.001
Carbohydrase	0.009	0.001	0.043	0.667	0.527	0.835	0.848	0.001	0.051	0.011	0.029	0.008	0.126	0.007
Corn type × carbohydrase	0.695	0.988	0.719	0.992	0.997	0.971	0.956	0.998	0.995	0.984	0.526	0.879	0.871	0.899

a-c Means in the same column with different superscript letter are significantly different by Tukey test (*P* ≤ 0.05).

1 Means (on DM basis) were obtained from 8 replicate cages of 7 birds each.

2 EE = ether extract.

3 Amylase = 80 kilo-Novo α-amylase units (KNU)/kg of feed.

4 Xylanase = 100 β-xylanase units (FXU)/kg of feed.

The semi-flint corn has a vitreous endosperm, providing it a hard aspect as well as a rounded and firm appearance. Semi-dent corn kernels typically have a flinty endosperm in the sides and a floury endosperm in the center. In waxy grains, the endosperm is composed almost entirely by amylopectin, but presents more beta-glucans. In the current study, there were differences on starch digestibility by broilers according to the corn endosperm, probably due to the starch composition and amylose:amylopectin ratio.

In the present study, corn-based diets supplemented with 80 KNU/kg amylase led to improvements on AME, AME_n_, IDE and digestibility of resistant and total starch (*P* ≤ 0.01). These results are in line with Stefanello et al. (2019), where increasing levels of α-amylase (0–160 FXU/kg) led to linear increases on nutrient digestibility and energy utilization of corn by broilers. Xylanase supplementation at 100 FXU/kg also resulted in improvements on AME, AME_n_, and IDE as well as on starch, DM, and EE digestibility. Stefanello et al. (2016) also observed that increasing levels of xylanase (0–200 FXU/kg) resulted in linear increases on nutrient digestibility and energy utilization of corn by 24-day-old broilers.

Corn diets supplemented with amylase + xylanase had improvements averaging 2.5, 2.1, and 3% for AME_n_, EE, and total starch digestibility, respectively (*P* ≤ 0.01). No differences were found in total tract metabolizability of Ca, P, N, and EE when corn diets were supplemented with carbohydrases (*P* > 0.05). In this field, there are few publications that analyzed energy utilization of corn separating the effects of soybean meal or other ingredients (Yegani and Korver, 2013; Tang et al., 2014; Stefanello et al., 2016, 2019).

The improvement on ileal digestibility of N observed in the present study when amylase and xylanase were supplemented agrees with Meng et al. (2005). The authors showed that exogenous carbohydrases can hydrolyze carbohydrate-protein complexes, facilitating the proteolysis of protein constituents of complex structures, leading to increases also in N digestibility. The decrease in endogenous losses of N and amino acids (**AA**) in the digestive tract has also been cited as a mechanism contributing to enzyme-induced increases in the ileal digestibility of AA (Cowieson and Ravindran, 2008), which has an additional focus on sustainable production systems.

Corn NSP content can be substantially high in the overall diet due to a high corn inclusion rate in corn-based diets. The highest digestibility and energy obtained with enzyme supplementation in the current study agrees with other researchers suggesting that exogenous carbohydrases increased energy availability by destroying some fractions of the cell walls of ingredients and enhancing starch digestion via increases in the endogenous amylase access to the starch granules in corn-soybean feeds (Schramm et al., 2021).

Yegani and Korver (2013), evaluating 3 different types of corn, stated that xylanase, xylanase + amylase + protease, or xylanase + β-glucanase supplementation had greater impact on digestibility by broilers fed corn with higher CP (9.5%), lower EE (3.7%), and intermediate starch (69.5%) composition compared to other 2 corn sources. Tang et al. (2014) reported an interaction between corn quality and enzyme supplementation for broilers, and the high-quality corn was seen to be an advantage in improving nutritional value when enzymes were used.

In conclusion, corn origin affected energy utilization and nutrient digestibility by broilers, and the effects of energy releasing carbohydrases supplemented to corn depend on their substrate differences. Corn classified as semi-flint presented the highest metabolizable energy and ether extract digestibility, whereas the semi-dent corn had the highest resistant starch digestibility for broilers. For this reason, the endosperm texture can also influence the energy values and nutrient digestibility of corn. Nevertheless, the majority of benefits on energy and nutrient digestibility of corn was obtained using the amylase and xylanase combination.

## References

[bib0001] AOAC International (2000).

[bib0002] Cowieson A.J. (2005). Factors that affect the nutritional value of maize for broilers. Anim. Feed Sci. Technol..

[bib0003] Cowieson A.J., Ravindran V. (2008). Effect of exogenous enzymes in maize-based diets varying in nutrient density for young broilers: growth performance and digestibility of energy, minerals and amino acids. Br. Poult. Sci..

[bib0004] Meng X., Slominski B.A., Nyachoti C.M., Campbell L.D., Guenter W. (2005). Degradation of cell wall polysaccharides by combinations of carbohydrase enzymes and their effect on nutrient utilization and broiler chicken performance. Poult. Sci..

[bib0005] Schramm V.G., Massuquetto A., Bassi L.S., Zavelinski V.A.B., Sorbara J.O.B., Cowieson A.J., Félix A.P., Maiorka A. (2021). Exogenous α-amylase improves the digestibility of corn and corn–soybean meal diets for broilers. Poult. Sci..

[bib0006] Stefanello C., Vieira S.L., Carvalho P.S., Sorbara J.O.B., Cowieson A.J. (2016). Energy and nutrient utilization of broiler chickens fed corn-soybean meal and corn-based diets supplemented with xylanase. Poult. Sci..

[bib0007] Stefanello C., Vieira S.L., Soster P., Santos B.M., Dalmoro Y.K., Favero A., Cowieson A. (2019). Utilization of corn-based diets supplemented with an exogenous a-amylase for broilers. Poult. Sci..

[bib0008] Tang S.H.D., Liu G., Nian F., Ru Y. (2014). Effects of maize source and complex enzymes on performance and nutrient utilization of broilers. Asian Australas. J. Anim. Sci..

[bib0009] Weurding R.E., Veldman W.A.G.V., van der Aar P.J., Verstegen M.W.A. (2001). Starch digestion rate in the small intestine of broiler chickens differs among feedstuffs. J. Nutr..

[bib0010] Yegani M., Korver D.R. (2013). Effects of corn source and exogenous enzymes on growth performance and nutrient digestibility in broiler chickens. Poult. Sci..

